# Supplementary material for: Lactiplantibacillus plantarum HY7718 Attenuates Renal Injury in an Adenine-Induced Chronic Kidney Disease Mouse Model via Inhibition of Inflammation and Apoptosis
Source: Int J Mol Sci. 2025 Oct 15;26(20):10052. doi: 10.3390/ijms262010052 (PMC12563546; doi:10.3390/ijms262010052)
Supplement: Supplementary file 1 [file ijms-26-10052-s001.zip › ijms-3911747-supplementary.pdf]

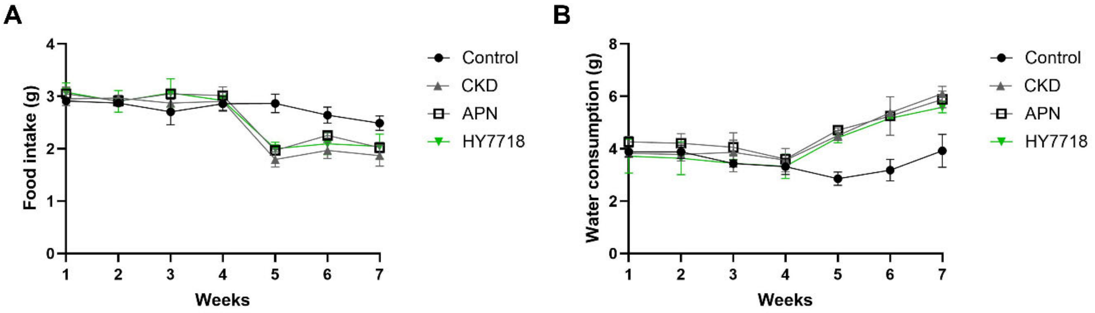

Figure S1. Dietary intake and water consumption. (A) Amounts of dietary intake (g), (B) water consumption (g) during animal experiments. Results are presented as the mean ± SD.
